# Elements that influenced immediate mother-neonate contact during the golden hour^*^

**DOI:** 10.1590/1980-220X-REEUSP-2022-0015en

**Published:** 2022-08-22

**Authors:** Bruna Rodrigues Monteiro, Valéria Gomes Fernandes da Silva, Anny Suelen dos Santos Andrade, Luciara Silva Machado, Erika Simone Galvão Pinto, Nilba Lima de Souza

**Affiliations:** 1 Universidade Federal do Rio Grande do Norte, Programa de Pós-Graduação em Enfermagem, Natal, RN, Brazil.; 2 Universidade Federal do Rio Grande do Norte, Programa de Graduação em Enfermagem, Natal, RN, Brazil.

**Keywords:** Mother-Child Relations, Delivery of Health Care, Labor, Obstetric, Obstetric Nursing, Infant, Newborn, Relaciones Madre-Hijo, Atención a la Salud, Trabajo de Parto, Enfermería Obstétrica, Recién Nacido, Relações Mãe-Filho, Atenção à Saúde, Trabalho de Parto, Enfermagem Obstétrica, Recém-Nascido

## Abstract

**Objective:**

To characterize the elements that influenced the immediate mother-neonate contact during the golden hour.

**Method:**

A cross-sectional observational study with a quantitative approach. A total of 105 parturient women hospitalized in two maternity hospitals with usual risk were observed. The instrument was based on Brazilian National Normal Childbirth Care Guidelines and World Health Organization good obstetric practices, totaling 36 questions. The analysis took place in a descriptive way using the Chi-Square Test for proportion comparison.

**Results:**

Of the parturient women, 2.8% (n = 3) experienced the golden hour, and 82.9% (n = 87), immediate contact between 1 and 5 minutes. In 85.7% (n = 90) of the group, there were no causes that contraindicated immediate contact. For 48.0% (n = 49) of participants, contact was re-established by the nursing staff within 31–60 minutes.

**Conclusion:**

Immediate contact during the golden hour had low hospital care compliance. Neonatal procedures that can be postponed predominated as influencing elements of the golden hour. The assistance observed in the birth rooms investigated reflects the need to reduce interventions in labor and birth.

## RESUMO

**Objetivo:**

Caracterizar os elementos que influenciaram no contato imediato entre mãe e bebê na hora dourada.

**Método:**

Estudo observacional transversal, com abordagem quantitativa. Foram observadas 105 parturientes internadas em duas maternidades de risco habitual. O instrumento teve como base as Diretrizes Nacionais de Assistência ao Parto Normal e as boas práticas obstétricas da Organização Mundial da Saúde, totalizando 36 questões. A análise ocorreu de forma descritiva mediante o Teste do Qui-Quadrado para comparação de proporção.

**Resultados:**

Das parturientes, 2,8% (n = 3) vivenciaram a hora dourada, e 82,9% (n = 87), o contato imediato entre 1 e 5 minutos. Em 85,7% (n = 90) do grupo, não houve causas que contraindicassem o contato imediato. Para 48,0% (n = 49) das participantes, o contato foi restabelecido pela enfermagem entre 31–60 minutos.

**Conclusão:**

O contato imediato na hora dourada teve baixa adesão na assistência hospitalar. Os procedimentos neonatais passíveis de serem adiados predominaram como elementos influenciadores da hora dourada. A assistência observada nas salas de parto investigadas reflete a necessidade de reduzir as intervenções no parto e nascimento.

## RESUMEN

**Objetivo:**

Caracterizar los elementos que influyeron en el contacto inmediato entre madre y bebé en la hora dorada.

**Método:**

Estudio observacional transversal con enfoque cuantitativo. Se observaron un total de 105 parturientas hospitalizadas en dos maternidades de riesgo habitual. El instrumento se basó en la guía para la atención del parto normal en Brasil y las buenas prácticas obstétricas de la Organización Mundial de la Salud, totalizando 36 preguntas. El análisis se realizó de forma descriptiva utilizando la Prueba Chi-Cuadrado para comparación de proporciones.

**Resultados:**

De las parturientas, 2,8% (n = 3) experimentó la hora dorada y 82,9% (n = 87), el contacto inmediato entre 1 y 5 minutos. En el 85,7% (n = 90) del grupo no hubo causas que contraindicaran el contacto inmediato. Para el 48,0% (n = 49) de los participantes, el contacto fue restablecido por el personal de enfermería dentro de los 31–60 minutos.

**Conclusión:**

El contacto inmediato en la hora dorada tuvo baja adherencia en la atención hospitalaria. Los procedimientos neonatales que pueden posponerse predominaron como elementos influyentes de la hora dorada. La asistencia observada en las salas de parto investigadas refleja la necesidad de reducir las intervenciones en el trabajo de parto y nacimiento.

## INTRODUCTION

A neonate’s first hour of life, called “golden hour”, requires health professionals to identify potential risks to neonates’ survival and the execution of practices based on scientific evidence taken as appropriate care, such as skin-to-skin contact (SSC) between mother and neonate, which acts as a recommended therapy^(1,2)^.

SSC promotes, for neonates, body temperature control, cardiorespiratory stability and reduced risk of hypoglycemia, with a consequent reduction in hospitalization time. For the mother, this interaction favors the establishment of a bond, the encouragement of breastfeeding by means of sucking, reducing, in the mother, the anxiety resulting from the gestational wait^(3)^.

In terms of recommendations, the World Health Organization (WHO), since 1996, treats this practice as a positive experience of care by the health staff during intrapartum^(4)^. Accordingly, the Brazilian Ministry of Health (MoH) guides encouraging SSC to parturient women, in order to minimize detachment and ensure contact immediately after childbirth^(5)^.

However, it is verified, from maternal reports, that, in the hospital environment, SSC is interrupted by the health staff to perform institutional routine procedures and this occurs automatically, not giving the opportunity to form a bond^(6)^. Regarding contact temporality, authors indicate as a practice that should be performed immediately after childbirth^(7)^, although there are no precise records in studies that show the time of SSC beginning, frequency and duration^(8,9)^.

There are scientific gaps that highlight the difficulties in starting and maintaining SSC during the golden hour, as well as the record of maternal and/or neonatal complications in intrapartum, in addition to records of structural elements assigned to professionals and childbirth care services.

Based on this, the present investigation has a pioneering character in the chronological identification and, at the same time, of elements that influenced SSC between mother and neonate during the golden hour, simultaneously, in two public maternity hospitals providing assistance to normal-risk childbirth. The study aimed to characterize the elements that influenced immediate contact between mother and neonate during the golden hour.

## METHOD

### Desig of Study

This is a cross-sectional observational study with a quantitative approach.

### Population

The population consisted of parturient women hospitalized in maternity wards of usual risk who evolved to vaginal birth. To determine the sample size, the finite sample calculation equation was used, considering the 95% confidence level and an estimated margin of error of 0.075. We adopted the expected prevalence of 0.40 for normal births and an average number of 290 births performed in both services in the period of three months, resulting in 105 parturient women.

### Local

The resarch was carried out in the prepartum, childbirth and postpartum (PCP) units of two maternity hospitals located in the city of Natal and Santa Cruz, state of Rio Grande do Norte, Brazil.

Both public maternity hospitals have multidisciplinary obstetric care and a structure favorable to good obstetric practices, represented by the PCP, in addition to being a reference in the care of pregnant women classified as at usual risk.

### Selection Criteria

The study included pregnant women at usual risk, with gestational age > 37 weeks, on the verge of birth. The study excluded pregnant women who evolved to surgical birth and those who, on the verge of birth, experienced an instrumentalized birth.

### Data Collection

Data collection occurred by convenience, from May to July 2019, by the researcher and three research assistants, after leveling. Participant selection took place from the admission of parturient women eligible for the study to PCP units, who accepted to participate in the research and, in follow-up, progressed to the second stage of normal birth.

For data collection, a structured instrument in the form of a form was used, based on the WHO recommendations and the MoH Brazilian National Normal Childbirth Care Guidelines^(4,5,10)^, in order to enable the identification of variables that portray the possible elements that influenced SSC between mother and neonate during the golden hour.

Thus, the form consisted of five axes: the first, aimed at characterizing the participants, with socioeconomic and obstetric data on parturient women; the second, referring to the characterization of contact during the golden hour, with general data of elements that influenced the practice; the third, representing influencing maternal elements, with the characterization of maternal procedures/complications; the fourth, characterizing the influencing neonatal elements, represented by neonatal procedures/complications; and the fifth axis, referring to the influencing structural elements observed in the assistance characterized by the lack of interprofessional staff communication and dyad transfer.

The instrument had 36 questions and was only applied after being tested, to suit the proposed objectives of the project, in a maternity hospital with the same care profile.

Altogether, four researchers participated in level training to use the instrument, and, later, the KAPPA test was performed to confirm coherence and skills. They were also instructed as to the inherent conduct of an observer^(11)^, in order to respect the parturient women’s hospital routine and space. The researchers followed participants’ birth and remained as observers on site throughout the period corresponding to the golden hour (one hour postpartum).

### Data Analysis and Treatment

The collected data were recorded and stored in an electronic spreadsheet and submitted to Statistical Package for the Social Sciences (SPSS), version 18, for analysis.

By characterizing the parturient women’s and neonates’ personal and socioeconomic profile and obstetric conditions, the respective frequency distributions were constructed, in addition to the characterization of contact and maternal and neonatal elements that delayed/compromised SSC between mother and neonate during the golden hour. To compare the percentages of the elements in the general distribution, the chi-square test was applied to compare the proportion. All conclusions considered a significance level of 5% (P < 0.05).

### Ethical Aspects

Before starting observation, parturient women were instructed about the research’s objective, potential risks and expected results, in order to ensure the right to privacy and confidentiality. After accepting the declared conditions, parturient women signed the consent terms, in two copies, authorizing the research, and the possibility of withdrawal, by the volunteers, at any stage of the research.

The research followed the ethical precepts, according to Resolution 466/12 of the Brazilian National Health Council, which deals with research involving human beings, after acceptance by the institutions and approval by the Research Ethics Committee (REC) of the *Universidade Federal do Rio Grande do Norte*, under Opinion 3,187,286, with approval in the first half of 2019. The authors informed the woman about the social contribution when participating in the study, anonymity and the possibility of withdrawing at any stage of this research.

## RESULTS

The study observed 105 parturient women, who evolved to vaginal birth, in two maternities located in northeastern Brazil.

After continuous observation of the care provided to mother and neonate in the birth room for 60 minutes, without interruption, the results were subdivided into: participant characterization; contact characterization during the golden hour; and maternal, neonatal and structural elements, respectively, that influenced the immediate mother-neonate contact.

The maternal and neonatal elements were categorized into procedures and complications. For the procedures, the care provided by the health staff in the birth setting and practices to prevent complications were considered. The complications were characterized as practices aimed at maternal and neonatal survival that required immediate intervention by the staff.

### Participant Characterization

The socioeconomic profile of parturient women showed that 66 (62.9%) were aged 19 to 30 years, 88 (83.8%) had a partner, 72 (68.6%) were brown and 61 (58.1%) had completed high school. As for origin, 65 (61.9%) came from the city where the birth took place and 83 (79%) had a family income of up to one minimum wage. In obstetric conditions, all parturient women had gestational age (GA) greater than 37 weeks and 56 (53.3%), with more than six prenatal consultations. Of these, 46 (43.9%) had no children, 59 (56.2%) desired pregnancy, 100 (95.2%) had no serological changes during pregnancy and 71 (67.6%) had no initial complications during pregnancy.

Regarding the knowledge of SSC immediately after birth, the same test showed that 78 (74.2%) reported lack ok knowledge of this right. The chi-square test, to compare the proportion, was significant in all socioeconomic and obstetric characteristics of parturient women (p-value < 0.05), except for the desire for pregnancy (p-value = 0.205), as shown in Table 1.

**Table 1.** Characterization of socioeconomic and obstetric profile of parturient women (n = 105) – Natal and Santa Cruz, RN, Brazil, 2019.

Variables assessed	Total		
	n	%	p-value
**Age**			
15 to 18 years	23	21.9	<0.001^1^
19 to 30 years	66	62.9	
31 to 45 years	16	15.2	
**Marital status**			
Without a partner	17	16.2	<0.001^1^
With a partner	88	83.8	
**Skin color**			
White	29	27.6	<0.001^1^
Black	4	3.8	
Brown	72	68.6	
**Education**			
Illiterate	1	1.0	<0.001^1^
Elementary school	37	35.2	
High school	61	58.1	
Higher education	6	5.7	
**Origin**			
Host city	65	61.9	0.015^1^
Adjacent city	40	38.1	
**Monthly income**			
None	3	2.9	
Up to 1 minimum wage	83	79.0	<0.001^1^
From 2 to 3 minimum wages	19	18.1	
**Gestational age**			
Term	103	98.1	<0.001^1^
Post-term	2	1.9	
**Number of prenatal consultations**			
Less than 6 consultations	22	21.0	<0.001^1^
6 to 9 consultations	56	53.3	
More than 9 consultations	27	25.7	
**Number of current children**			
No children	46	43.9	<0.001^1^
One child	29	27.6	
Two children	14	13.3	
Three or more children	16	15.2	
**Desired pregnancy**			
No	46	43.8	0.205^1^
Yes	59	56.2	
**Serological alteration during pregnancy**			
No serological alteration	100	95.2	<0.001^1^
Toxoplasmosis	4	3.8	
Syphilis	1	1.0	
**Initial complications in pregnancy**			
Absent	71	67.6	<0.001^1^
Urinary tract infection	26	24.8	
Depression	1	1.0	
Alcohol and tobacco	2	1.9	
Hemorrhage	1	1.0	
Anemia	1	1.0	
Arbovirus	2	1.9	
Gestational hypertension	1	1.0	
**Location of skin-to-skin contact information**			
None	78	74.3	<0.001^1^
Maternity	3	2.9	
Prenatal care	13	12.4	
Previous birth	3	2.9	
Multimedia/family/company	8	7.6	

Note: ^1^p-value of the chi-square test for proportion comparison.

As for the neonates, the information collected during the first postpartum hour showed that 86 (81.9%) had 1-minute APGAR between 8–10, and 96 (91.4%), 5-min APGAR between 8–10. In 89 (84.7%) cases, there was no record of malformation, and 24 (22.8%) neonates had their weight recorded in the range of 2.5–4.5 kg. During observation time, there was no record regarding birth weight in 81 (77.2%) cases or record of malformation in 15 (15.3%) records of neonates in the first two hours of life. The proportion comparison test was significant in all assessed elements (p-value < 0.05), indicating that the profile described is, significantly, the most frequent in the assessed group.

### Golden Hour Contact Characterization

Table 2 shows the immediate SSC observation characteristics. It appears that 90 (85.7%) of neonates were placed in SSC soon after birth, in 62 (59.0%) cases an obstetrician was responsible for this action, and SSC duration for 87 (82.9%) of them did not exceed five minutes, with a quantitative of 59 (56.2%) dyads who experienced skin-to-cloth contact.

**Table 2.** Characterization of immediate contact and influencing elements (n = 105) – Natal and Santa Cruz, RN, Brazil, 2019.

Variables assessed	Total		
	n	%	p-value
**Beginning of immediate contact (minutes)**			<0.001^1^
Immediately	90	85.7	
5–20 minutes	8	7.6	
>60 minutes	7	6.7	
**Responsible for beginning contact**			
No initial contact	7	6.7	<0.001^1^
Pediatrician	5	4.8	
Nursing	31	29.5	
Obstetrician	62	59.0	
**Elements that influenced the beginning of immediate contact**			
Absence of elements	90	85.7	<0.001^1^
Maternal complications/procedures	0	0	
Complications in the neonate	7	6.7	
Procedures on the neonate	6	5.7	
Maternal refusal	2	1.9	
**Immediate contact duration (minutes)**			
Immediate contact not performed	7	6.7	<0.001^1^
1 to 5 minutes	87	82.9	
6 to 59 minutes	8	7.6	
60 minutes	3	2.8	
**Type of immediate contact**			<0.001^1^
Not performed	7	6.7	
Skin-to-cloth contact	59	56.2	
Skin-to-skin contact	39	37.1	
**Elements that influenced immediate contact duration**			
Absence of elements	11	10.5	<0.001^1^
Maternal procedures	6	5.7	
Procedures on the neonate	71	67.6	
Complications in the neonate	16	15.2	
Structural elements	1	1	
**Responsible for completing immediate contact**			
Absence of someone responsible	11	10.4	<0.001^1^
Pediatrician	85	81.0	
Nursing staff	3	2.9	
Obstetrician	4	3.8	
Companion/father	2	1.9	

Note: ^1^p-value of the chi-square test for proportion comparison.

As for influencing elements in immediate contact duration, it was observed that, in 71 (67.6%) births, the performance of procedures on the neonate that could be postponed prevailed and, in 85 (81%), the pediatrician acted as the main responsible for SSC interruption.

Table 2 shows 11 neonates with the absence of elements that influenced immediate contact duration as well as the absence of those responsible for finalizing immediate contact. The total of both variables corresponds to the following groups of neonates: three neonates, who experienced SSC for 60 minutes without interruption; seven neonates, who did not experience immediate contact at any time; and a neonate, who was delayed in initiating immediate contact, but remained in contact with the mother until the end of the golden hour.

As for late contact and influencing element characterization, according to Table 3, it is verified that 49 (48.0%) dyads had SSC reestablished in the interval of 31–60 minutes, beginning the late contact with the mother. In 55 (53.9%) of cases, the procedure in the neonate acted as an influencing element in the delay of returning the neonate to the mother’s lap.

**Table 3.** Characterization of late contact and influencing elements (n = 102) – Natal and Santa Cruz, RN, Brazil, 2019.

Variables assessed	Total		
	n	%	p-value
**Beginning of late contact (minutes)**			
Up to 10 min	17	16.7	<0.001^1^
11 to 20 min	25	24.5	
21 to 30 min	11	10.8	
31 to 60 min	49	48.0	
**Elements responsible for the delay to return the neonate**			
Absence of elements	3	2.9	<0.001^1^
Procedures on the mother	17	16.6	
Procedures on the neonate	55	53.9	
Structural elements	10	9.8	
Complications in the mother	0	0.0	
Complications in the neonate	14	13.7	
Other causes (maternal refusal and scabies)	3	2.9	
**Responsible for late contact**			
Not applicable	34	33.3	<0.001^1^
Pediatrician	16	15.7	
Nursing staff	44	43.1	
Companion	5	4.9	
Puerperal woman	3	2.9	

Note: ^1^Chi-square test for proportion comparison.

As responsible for the beginning of late contact, the nursing staff stood out in 44 (43.1%) cases, followed by the category does not apply in 34 (33.3%) cases, as shown in Table 3. This variable corresponds to neonates who did not experience late contact, that is, neonates who were experiencing SSC uninterruptedly (n = 3), who did not experience late contact because of neonatal resuscitation (n = 7) and those (n = 24) who remained in the crib until completing one hour of life, with no professional/guardian to encourage the neonate’s return to the mother’s lap after the assistance provided by the health staff.

### Influencing Maternal Elements

The maternal influencing elements were categorized in detail in maternal procedures and complications. Regarding the procedures, no maternal procedure was observed that would compromise the beginning of immediate contact or maternal complications in the first hour of a neonate’s life. Meanwhile, four procedures were identified that compromised immediate contact duration and the neonate’s return to contact during the golden hour. Despite the existence of maternal procedures, it appears that the absence of procedures influencing immediate contact duration and the absence of procedures influencing the neonate’s return to SSC prevailed, as shown in Table 4.

**Table 4.** Distribution of maternal procedures influencing immediate contact duration and late neonate’s return (n = 105) – Natal and Santa Cruz, RN, Brazil, 2019.

Maternal procedures assessed	Total		
	n	%	p-value
**Influencing procedures in immediate contact duration**			
Absence of influencing procedures	100	95.0	<0.001^1^
Perineum repair	2	2.0	
Bed hygiene	1	1.0	
Exit of the stool to bed	1	1.0	
Meal	1	1.0	
**Procedures that influenced the neonate’s return to skin-to-skin contact**			
Absence of influencing procedures	88	83.8	<0.001^1^
Birth canal revision	5	4.8	
Uterine massage	2	1.9	
Bed hygiene	7	6.7	
Meal	3	2.9	

Note: ^1^p-value of the chi-square test for proportion comparison.

It is possible to verify maternal care procedures (birth canal revision, perineal repair) that do not contraindicate and can occur concomitantly with immediate SSC practice, in addition to service characteristics that acted as elements that made it difficult to encourage uninterrupted SSC during the golden hour.

### Influencing Neonatal Elements

The neonatal elements that influenced were categorized into groups of procedures, due to neonates’ experience in more than one neonatal procedure, in addition to neonatal complications.

At all times, neonate procedures were highlighted as an element responsible for immediate contact interruption, delay in returning to SSC and late beginning of SSC. Regarding the procedures in the neonate responsible for immediate contact interruption, 71 (67.6%) of neonates experienced, on average, 7.5 procedures in the first hour of life. In all, 22 procedures were responsible for immediate contact interruption, as observed in Table 5.

**Table 5.** Distribution of neonatal procedures that influenced skin-to-skin contact interruption in the first hour of life (n = 71) – Natal and Santa Cruz, RN, Brazil, 2019.

Neonatal procedures assessed	Total	
	n	%
Airway aspiration (procedure)	69	13.0
Clamping	66	12.3
Physical exam and practical class for health students	64	12.0
Vitamin K	47	8.8
Nasogastric tube passage	38	7.2
Identification wristband placement	35	6.6
Drying the neonate	35	6.6
Rectal probe passage	34	6.4
Field swap	24	4.5
Cap	21	4.0
Photography	21	4.0
Anthropometric measurements	18	3.4
Weighing	18	3.4
Plantar identification	11	2.1
Health education/navel cleansing	7	1.3
Maternal serology	6	0.9
Clothing	5	0.8
Diaper	4	0.6
Eye protection	3	0.6
Heating neonates in the crib	2	0.4
Latch on assessment	2	0.4
Butterfly touch massage	1	0.2

Regarding the complications observed in the neonates, seven (6.7%) neonates experienced the neonatal resuscitation protocol, being the only complication present in the initial encouragement of SSC. Immediate contact interruption occurred in six (37.5%) cases, neonatal resuscitation and significant meconium in five (31.3%), respiratory distress in four (25.0%). One (6.3%) was related to decreased tonus, making a total of four elements responsible for interruption in the group of complications. Meanwhile, for the delay in the neonate’s return to contact, three types of complications were identified: 14 (82.4%) indications of Hood; two (11.8%) neonate transfers to the Intensive Care Unit; and one (5.9%) monitoring of vital signs.

The golden hour, which corresponds to one hour of uninterrupted SSC between mother and neonate, in the first hour postpartum, was recorded in only three (2.8%) dyads of the study population, while seven (6.7%) dyads did not experience SSC at any time in the first hour of life.

### Influencing Structural Elements

Regarding the profile of maternity care, using the Chi-Square Test to compare the proportion, 44 (41.9%) births were significantly observed in the morning shift (p-value = 0.004) and in 19 (18.1%) cases, Tuesday stood out, despite the similarity between the other days (p-value = 0.879).

It is important to note that, during the observation, the predominance of variables called structural elements was detected, which influenced the duration and neonate’s return to contact with the mother. In immediate contact duration interruption, Table 2 evidenced one (1%) structural element that, in this case, corresponds exclusively to the dyad transfer.

Meanwhile, to return the neonate to the beginning of late contact, Table 3 depicts 10 (9.8%) elements associated with structural factors, which corresponds to six (60%) dyad transfers to another sector and four (40%) cases of lack of interprofessional staff communication to encourage late contact.

## DISCUSSION

In our study, immediate contact duration during the golden hour was 1 to 5 minutes in 82.9% of cases, and only 37.1% of neonates experienced SSC. In 67.6% of cases, immediate contact interruption to perform procedures on the neonate was observed, which may show the fragility of care practice in the birth room, in the face of procedures, in an underdeveloped country.

Immediate SSC during the golden hour is a practice with ministerial recommendations in Brazil and worldwide. The association of SSC with the golden hour is related to the release of hormones at the time of childbirth, such as oxytocin, and, at the same time, to the elimination of stress hormones generated in labor^(12)^, calming them, in addition to favoring dyad recognition and sensitization^(7)^.

The practice is widely recommended, especially when one has a normal birth without complications and the neonate has good fetal vitality^(13)^. The research data show that the dyads were considered to be at usual risk, without complications in labor/birth that were responsible for the separation during the golden hour, thus favorable to the execution of SSC.

Regarding prenatal care, the records show the prevalence of six to nine prenatal consultations without proper guidance to the mother regarding SSC both in prenatal care and in the maternity responsible for perinatal care. Although the number of prenatal consultations (>4) is expected to favor immediate contact^(8,14)^, the mothers perceived a low level of knowledge regarding the right to SSC during the golden hour.

Prenatal care has the mission of favoring the exchange of experiences and knowledge between professionals and pregnant women about good obstetric practices^(15,16)^. However, socioeconomic disadvantage, as evidenced in this research, based on color, age group, income and education, has been a factor of stigma and discrimination in the care of pregnant women in prenatal care and maternity^(17)^. This fact requires the professional to understand the reality of submission of women to the obstetric model offered by the Unified Health System (SUS – *Sistema Único de Saúde*)^(18)^.

Although most women experienced SSC immediately after birth, it is worth highlighting the early interruption and late return, with no record of maternal complications and few neonatal complications that would contraindicate this practice. This reduced duration was evidenced in another investigation, in which 73% of dyads were separated before 60 minutes^(13)^.

With regard to structural elements and, more precisely, the sector organization, the literature shows the reduced number of human resources for childbirth care^(1)^, as well as the demand for obstetric beds higher than that offered in the institution^(5)^, causing the dyad to be filled and transferred between sectors.

In the present study, the results show again the problem of capacity, not differentiating between maternity hospitals in Brazil; however, despite the sufficient number of employees, the study points out as a structuring element of causality the lack of interprofessional communication between the staff for co-responsibility in encouraging late contact, since the practice can and should be encouraged by the entire staff^(4,5)^.

The performance of SSC is conditioned, in most cases, to encouraging breastfeeding, being identified in the neonatal elements the postponement of contact encouragement due to absence of maternal serology. Such conduct is not different from other studies that perform the direct association of SSC with breastfeeding, after initial medical assessment of neonates, within the first five minutes of life, and, in cases of neonatal complications, after 24 minutes of life^(9,19,20)^.

Despite the positive influences of SSC for exclusive breastfeeding (EBF), it should not be conditioned to just one fact, so as not to interrupt the bond between mother and neonate, when contraindicated or when the mother is unable to breastfeed^(9)^, due to numerous benefits of SSC in the birth room and in the first hour of a neonate’s life.

In this investigation, the existence of non-recommended practices was evidenced, practices that could be postponed or readapted in the care provided to the neonate, such as drying the neonate in a warm crib, performing skin-to-cloth contact, as well as invasive procedures. Investigations point to the prioritization of procedures inherent to the institutional routine in the face of SSC without the mother’s consent, in addition to the execution of skin-to-cloth contact and procedures that are no longer recommended^(4,6,21)^.

In the recommendations of good practices in dyad care, the WHO portrays the need for early SSC, delaying bathing by up to 24 hours, and prophylaxis of hemorrhage with vitamin K after the first hour of life^(4)^. The Brazilian National Normal Childbirth Care Guidelines and the WHO, in general, recommend identifying maternal and/or neonatal complications based on good practices, prioritizing, as far as possible, SSC between mother and neonate^(4,5,10)^.

Evidence of inappropriate conduct for completing the golden hour in its entirety, in both maternity hospitals investigated, requires attention from the obstetric and neonatal care staff, with a closer look at the recommendations of good practices for childbirth and birth^(4)^.

In this study, the nursing staff acted as responsible for the neonate’s return to SSC, although these professionals did not help in immediate contact and in encouraging the golden hour duration. It is noteworthy that obstetric nurses act as the main fosters of contact between mother and neonate after birth^(22,23)^.

Finally, the low prevalence of the golden hour and pregnant women’s low knowledge of their right to request SSC stand out. On the other hand, there is a predominance of procedures performed by health professionals in the care of mothers and neonates. The lack of knowledge could be evidenced in tertiary level hospitals, in which 82.2% of 164 mothers investigated were unaware of SSC practice^(24)^. As facilitators of SSC practice, it is evident that the knowledge and empowerment of pregnant women in prenatal care about SSC act as a basis for encouragement, in addition to the presence of a companion during childbirth^(22–25)^ and health staff surveillance focused on good obstetric practices^(6)^.

We identified barriers facing research investigators in the investigated institution and in the study population as limitations of this study. Concerning the institution, we evidenced distrust on the part of health professionals, while, from the researchers’ point of view, the number of members was reduced to remain in maternity hospitals on duty, not being possible to observe simultaneous births. As for the study population, the absence of uninterrupted SSC during the golden hour. Such limitations make it impossible to perform an association test, which suggests that the results do not generalize the reality of other health services. However, they guide the development of investigations with the same study design.

The study brings relevance to good practices in childbirth and birth in hospital units, emphasizing the importance of obstetric nursing as a facilitator in encouraging contact between mother and neonate during the golden hour and, for public health, the appreciation of public policies for childbirth care. It is worth reinforcing the importance of inserting the researcher in the care practice scenario, favored by this study, which allowed us to observe elements that intervene in SSC encouragement, correctly and uninterruptedly, during the golden hour.

## CONCLUSION

The present investigation acted as a pioneer in the direct and simultaneous observation of the care provided by the health staff, at two public maternity hospitals, encouraging immediate SSC during the golden hour, without interruption, with no intervention by the researchers in the care provided in the birth room. The study made it possible to detect the applicability, duration and elements that influenced this action, scientifically proven as good obstetric practice, whose observation period started at the moment of birth and segmented by one postpartum hour (golden hour).

In this scenario, we identified low compliance by maternity hospitals to encouraging SSC during the golden hour and the existence of adverse elements, mostly without obstetric or neonatal justification that had an indication for the dyad’s survival. The institutionalization of childbirth and birth, professionals’ active predominance to the detriment of parturient women, neonatal procedures that can be postponed and lack of knowledge of SSC during the golden hour were clearly perceived, according to scientific recommendations.

Thus, we concluded that elements that influenced immediate SSC encouragement are related to the hospital routine prioritization, disregard of the benefits for mother-neonate bond, lack of women’s role in childbirth and appropriation of professionals in childbirth and birth, without considering good obstetric practices, which suggests the need to readjust professional assistance in PCP beds.

It is worth noting that routine and institutionalized procedures, as well as professionals’ addictive behaviors, compromise good obstetric practices, proven beneficial to mothers and neonates, and that this fact violates women’s reproductive rights and good practices for childbirth, which includes SSC during the golden hour.

## Financial support

The present work was carried out with the support of the Coordination for the Improvement of Higher Education Personnel – Brazil (CAPES – *Coordenação de Aperfeiçoamento de Pessoal de Nível Superior*) – Financing Code 00.
